# Agricultural intensification in Lake Naivasha Catchment in Kenya and associated nutrients and pesticides pollution

**DOI:** 10.1038/s41598-024-67460-5

**Published:** 2024-08-09

**Authors:** Joel Onyango, Nzula Kitaka, J. J. A. van Bruggen, Kenneth Irvine, John Simaika

**Affiliations:** 1 IHE Department of Water Resources and Ecology, IHE Delft, Institute for Water Education, Westvest 7, P.O. Box3015, 2601DA Delft, The Netherlands; 2 Egerton University, Njoro, Kenya; 3 Aquatic Ecology and Water Quality Management, Wageningen University, P.O. Box 47, 6700AA Wageningen, The Netherlands; 4 African Centre for Technology Studies (ACTS), P.O. Box 45917, 00100 Nairobi, Kenya; 5 Stellenbosch University, Private Bag X1, Stellenbosch, South Africa

**Keywords:** Environmental chemistry, Limnology, Wetlands ecology

## Abstract

Investments in agricultural intensification in sub-Saharan Africa aim to fulfill food and economic demands. However, the increased use of fertilizers and pesticides poses ecological risks to water bodies in agricultural catchments. This study focused on assessing the impact of agricultural intensification on nutrient and pesticide pollution in the L. Naivasha catchment in Kenya. The research revealed significant changes in the catchment’s agricultural landscape between 1989 and 2019, driven by intensified agricultural expansion. As a result, nutrient and pesticide emissions have worsened the lake’s trophic status, shifting it towards hypereutrophic conditions. The study found a weak relationship between total nitrogen (TN) and sum dichlorodiphenyltrichloroethane (∑DDT), indicating that an increase in TN slightly predicted a reduction in ∑DDT. Analysis also showed potential phosphorus (P) limitation in the lake. Additionally, the observed ratio between dichlorodiphenyldichloroethane and dichlorodiphenyldichloroethylene (DDD:DDE) and (DDE + DDD):DDT ratios suggest recent use of banned DDT in the catchment. The study concludes that the transformation of L. Naivasha landscape shows unsustainable agricultural expansion with reduced forest cover, increased croplands, and increased pesticide contamination. This reflects a common issue in sub-Saharan Africa, that sustainable catchment management must address, specifically for combined pollutants, to support water quality and achieve the SDGs in agriculture.

## Introduction

Rapid population growth and development aspirations in sub-Saharan Africa (SSA) has led to increased investment in agricultural intensification to meet food and economic demands^1^. However, this intensification is hindered by low soil nutrient content, rapid soil-nutrient depletion^2,3^, and the presence of pests and diseases that reduce crop yields^4^. These challenges have driven increased application of fertilizers and pesticides^5,6^ aimed at enhancing productivity. Agricultural activities used over 300,000 metric tons of fertilizers in Kenya in 2019. In 2021 there was a net increase in fertilizer imports of 500 kilotonnes. Phosphate-based (150,000 metric tons), and nitrogen-based (140 metric tons) fertilizers were the most sought-after fertilizer components, an indication of N-limitation in agricultural production systems in Kenya^7^.On the other hand, pesticides imports in Kenya in 2022 was valued at USD 183million^8^, with net importation of USD 163 million^8^, increasing from USD 72 million in 2020 at a volume of 3068 tonnes of pesticides^9^. Agricultural intensification has the potential to increase nutrient and pesticide emission, and associated degradation of aquatic ecosystem functions^10–12^.

Nutrient enrichment can lead to eutrophication, increasing the risk of harmful algal blooms in lakes and aquatic ecosystem poisoning^13–16^. The primary nutrient drivers for eutrophication are nitrogen and phosphorus. These nutrients are often derived from human activities such as agriculture, sewage discharge, and industrial processes, therefore increasing nutrients loads resulting to extensive growth of algae and other aquatic flora^17,18^. In this study, agricultural-mediated eutrophication is the main area of focus, cognizant of complexities that would be derived from other human activities. Eutrophication disrupts ecosystem structure and function including, in some instances, direct toxicity to fish as a result of the production of cyanobacteria toxins that harm fish^10,18^.

Pesticide residues affect the physiology and ecology of aquatic biota, including poisoning organisms, resulting in a loss of local flora and fauna diversity^19,20^. Highly persistent pesticides such as dichlorodiphenyltrichloroethane (DDT), and hexachlorocyclohexane (HCH) used in crop protection, are readily adsorbed to soils and sediments, which can act both as sinks and as long-term sources of exposure affecting organisms^21^. Onyango et al.^22^ reported that 59% of the pesticides by active ingredients applied within the L. Naivasha catchment were moderately to extremely toxic, with 22% of the applied pesticides of international concern because of their high toxicity and persistence in the environment^22^. Balancing the goals of agricultural intensification with ecosystem protection is a key issue, also requiring effective monitoring of pesticide and fertilizer use and their leaching into water bodies. Collectively, a better understanding of the combined effects of nutrient enrichment and pesticide exposure is needed. This study aimed to determine the implication of agricultural intensification on nutrients and pesticide pollution in the L. Naivasha catchment, where both surface and groundwater resources have been heavily exploited^23^. The study focused on surface waters owing to higher contact area with overland nutrients and pesticides emission and higher magnitude of pollution compared with ground water in L. Naivasha catchment. The distribution and potential ecosystem degradation associated with combined nutrients and pesticides in the surface waters of the catchment, and their relation to land use changes, have not been explored previously. The catchment is renowned for horticulture and floriculture, which are important economic activities^24^ and contribute to nutrients and pesticides emission^24^. By investigating sources of nutrient and pesticide contamination in the catchment and potential impact on the aquatic ecosystems, the study aimed to contribute information for water quality management within agricultural catchments relevant to sustainable intensification in sub-Saharan Africa.

Our study aimed to answer four questions: (1) is agricultural intensification observable in the Lake Naivasha catchment?; (2) is the intensification related to the nutrients and pesticides emissions to surface waters ?; (3) is there a demonstratable impact of nutrient and pesticide emissions on the ecology of the recipient water bodies?; and, (4) what are the management options for intensification mediated nutrient and pesticides emissions?

## Materials and methods

### The Lake Naivasha catchment

The L. Naivasha catchment covers an area of 3400 km^2^ in the central Rift Valley region of Kenya, spanning Nakuru and Nyandarua counties^25^. It is the second largest freshwater lake in Kenya, with an approximate surface area of 100 square kilometers^26^. The lake is designated as a Ramsar site, recognized internationally for its ecological importance. The primary water sources for L. Naivasha are three main rivers: the R. Malewa, R. Gilgil, and R. Karati^26,27^. The catchment has a semi-arid climate, with mean monthly temperatures ranging from 15.9 to 17.8 °C^28^. The rainfall pattern in the region is bimodal, with the “long rains” typically occurring from April to May/June and the “short rains” from October to November^24^.

The upper reaches of the catchment are characterized by smallholder farming involving the cultivation of maize, beans, and vegetables^29^. The mid reaches comprise mixed farming, characterized by large scale livestock ranches, previously documented to be an ecological threat to aquatic life^30^. The catchment’s lowlands are a major horticulture region, responsible for over 70% of the cut-flower export from Kenya especially prevalent in the 50 km^2^ adjoining the lake in the north-east of the lake^30^. The contribution of these agricultural activities to water quality in the L. Naivasha catchment is not clearly understood.

### Site selection

**Figure 1:**
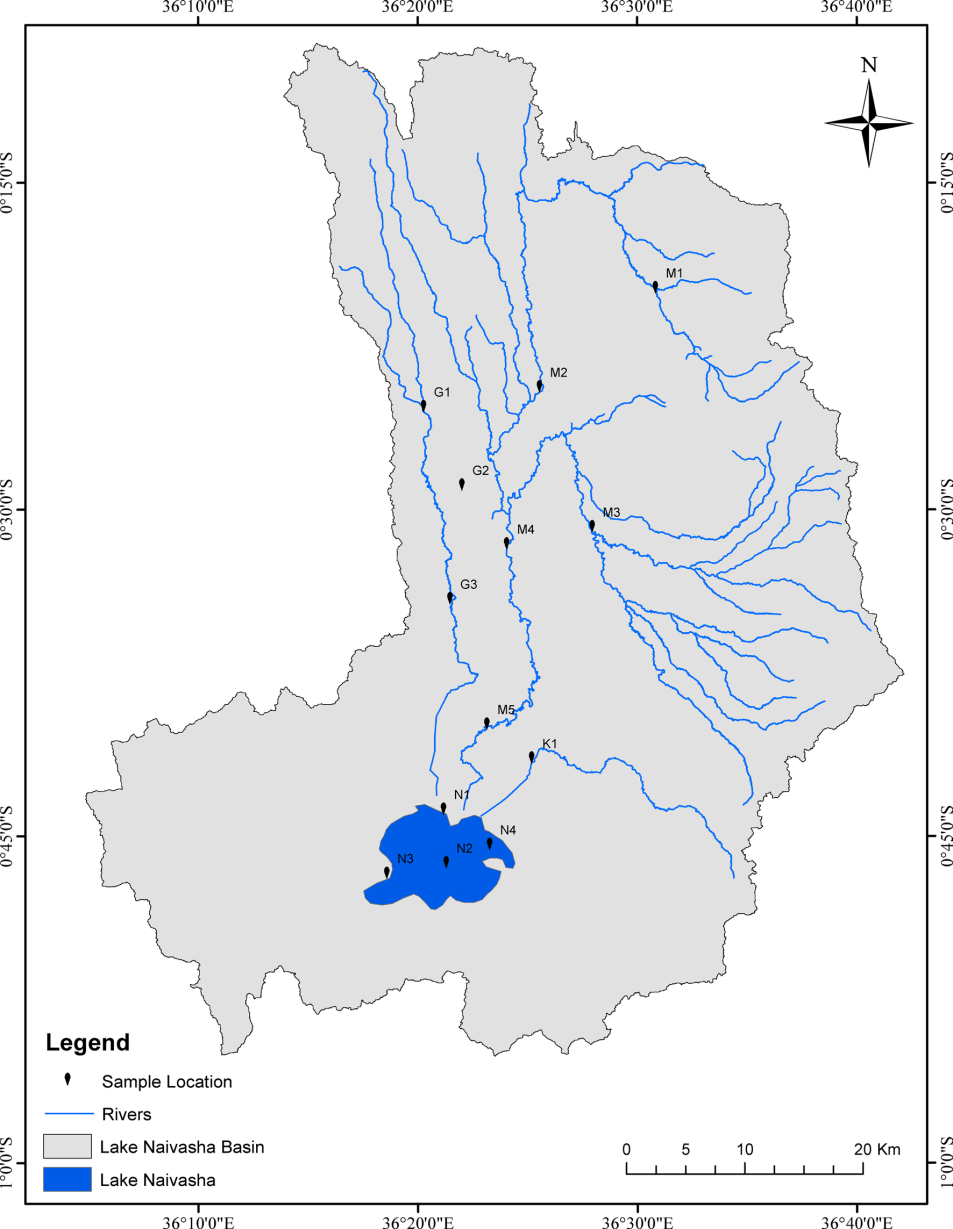
Lake Naivasha catchment and sub-catchments, Kenya. Sampling sites are: G1 = Kahuho, G2 = Little Gilgil, G3 = Gilgil Highway Bridge, M1 = Wanjohi, M2 = upper Malewa, M3 = Turasha, M4 = Bush Ventures, M5 = Malewa Highway Bridge, K1 = Karati Highway Bridge, N1 = River Mouth, N2 = Mid-lake, N3 = Hippo Point, N4 = Crescent.

Thirteen sampling stations were selected along R. Malewa, R. Gilgil and R. Karati, and in L. Naivasha (Figure 1). The sampling stations were chosen to represent different geographical areas within the catchment to assess water quality and the impact of nutrients and pesticides emission to ecology of the recipient surface water bodies in the catchment. Wanjohi (M1) and upper Malewa (M2) sites are in the upper reaches of the R. Malewa catchment, Malewa Bush Ventures (M4) in the mid reaches, and Malewa Highway (M5) in the lower reaches. The Turasha site (M3) is a tributary to the R. Malewa running from the north-east, before entering the Turasha dam. Kahuho site (G1) is in the upper part of the R. Gilgil catchment, while Little Gilgil site (G2) is a tributary to the main channel. The Gilgil Highway site (G3) is situated in the lower part of the catchment below the Gilgil dam. The only site along the R. Karati—the Karati Highway Bridge site (K1), is in the lower reaches of the river. The L. Naivasha sites include the River Mouth site (N1) near where the R. Gilgil and the R. Malewa enter the lake, the Mid-lake site (N2) intended to capture the conditions of the lake as a whole, the Hippo point site (N3) along the south-western lake shore area and far away from the riverine inputs, and the Crescent site (N4), north-east and close to the farms on the shores of the lake.

### Sampling

Monthly sampling was carried out between April and August 2015. In the river sites, three surface water samples were collected from 5 cm below the water surface from downstream-to-upstream encompassing, where present, riffle, run, and pool habitats within a 100 m reach. In the lake, water samples were collected using a Schindler sampler in the upper 5–30 cm, mid 3–4 m, and bottom 5–7 m depths. At each river reach, and lake sampling area, water samples were integrated into one consolidated sample. Each integrated sample was placed in an acid-washed 500 ml polythene bottle for nutrient analysis and in a 2.5 L dry amber glass bottle, pre-washed with distilled water followed by ethanol rinsing, for pesticides analysis. Water samples were transported in a cool box with icepacks to, respectively, the Egerton University Aquatic Sciences laboratory for nutrient analysis, and to the University of Nairobi Pesticide Research Laboratory for pesticides residues analysis.

### Laboratory analysis

In the laboratory, 300 ml of the unfiltered water was used to determine total nitrogen (TN) and total phosphorus (TP) concentrations in triplicate. TN was determined using the micro Kjeldahl method and TP determined through persulphate digestion followed by the ascorbic acid method^31^. Total suspended solids (TSS) was determined using the gravimetric method^31^.

Our study focused on residue concentrations of the pesticides HCH group (α-HCH, β-HCH, γ-HCH, δ-HCH), cyclodiene group (Heptachlor, Heptachlor Epoxide, α-endosulfan, β-endosulfan, endosulfan sulphate, endrin, endrin aldehyde, aldrin, dieldrin, and methoxychlor), and DDT group (pp-DDE, pp-DDD, pp-DDT). Pesticide residues in the water samples were extracted in triplicate using Liquid–Liquid extraction method following the UNEP-POP protocol^32^. The extraction was done according to the German Method (DIN EN ISO 6468: 1997) for water quality—determination of organochlorine pesticides, polychlorinated biphenyls and chlorobenzenes—gas chromatographic method after liquid–liquid extraction (ISO 6468: 1996, EN ISO 6468: 1996) using petroleum ether (DIN EN ISO 6468: 1997 section “Nutrients and pesticides emission in the catchment”).

Each sample was homogenized by shaking and then put into a 2 L separatory funnels. Next, 30 mL of petroleum ether were added to the sample. The separatory funnel was then put into a shaker for 10 min at 110 r.p.m. The suspension was left to settle for 15 min. The organic solvent phase and the water phase were separated by draining the water phase into the original water sample bottle. 25 ml of the organic solvent was then recovered from the separatory funnel into the concentration funnel. The water phase that was drained into the original water sample bottle was homogenized and then put back into the 2 L separatory flask. The extraction was repeated twice using 20 mL of petroleum ether, shaken for 10 min at 110 r.p.m. The solution was then left to settle for 15 min. The water phase was then drained into the original water sample bottle, and 20 mL of the organic solvent recovered from the separatory funnel drained into the concentration funnel. Each time the water phase that was drained into the original water sample bottle was homogenized and then put back into the 2 L separatory funnel. A total of approximately 65 mL of the organic solvent was recovered. The organic solvent in the concentration funnel for the water samples was concentrated to about 0.5 ml through liquid nitrogen gas mediated evaporation. An additional 0.5 ml of petroleum ether was used to clean the concentrating funnel of any attached active substances and then concentrated to 1 mL final volume.

The analysis of the pesticides was performed using an GC/MS using Full Scan and Selected Ion Monitoring using the Thermo Scientific DSQ™ Quadrupole. The samples and standards were injected using a programmable temperature vaporization large volume injector (PTV LV) with a cold solvent split injection of 5 µL. The PTV LV injector was configured with a salinized glass liner with a small wisp of saline-treated glass wool. The sample was injected at 50 °C for 6 s and then increased to 90 °C for 6 more seconds with the split vent open to evaporate the solvent. The split vent was held at an initial temperature of 50 °C, then closed for the pesticides to be thermally transferred into the analytical column, at 275 °C for one minute.

### Quality assurance and control

For quality assurance, spiked water samples were used to determine the recovery rates for each pesticide residue. Each of the pesticides under investigation was added in standard concentrations and processed using the same procedure as the field samples. Additionally, distilled water was used as blanks and incorporated with external standards to determine the detection limit of the pesticides investigated. A procedural blank was run in parallel with the samples, in a manner identical to the samples. Samples and blanks were spiked with the recovery standard solution mix prior to solvent extraction to monitor methodological losses. Recoveries of between 50 and 150% were accepted. The limits of detection (LODs) for the specific chemicals were estimated by the signal to noise ratio (3:1). The precision was estimated for each specific chemical as the relative standard deviation of the recoveries. Instrument calibration for the specific pesticides were done from 20 to 500 µg/L in acetone. The percent recoveries ranged from 70 to 95% while limit of detection ranged from 0.0011 to 0.0036 µg/L (more details in Supplementary Table 1) indicating that the sample processing provided reliable concentrations.

### Assessment of agricultural intensification

Satellite images from Landsat 4 (1989), Landsat 7 (1999), Landsat 5 (2009) and Landsat 6 (2019) were used to estimate and classify land use land cover (LULC). The satellite data was acquired from USGS: Earth Explorer for path169, row 60; path 168, row 61; path 169, row 60 and path 169 row 61. Catchment delineation was done using ArcGIS Version 10.3. Sub-catchment delineation was performed using predetermined pour-points for sampling. LULC classification was done using ENVI Version 5.1. Unsupervised classification was first performed using IsoData and K-means algorithms with a set of 10 classes. The classes were then used to define regions of interest (ROIs) for supervised maximum likelihood classification. The classes selected were generated from unsupervised classification and from previous studies conducted in the area^33^. Training samples were then selected, and the LULC classes divided into six types: open water, broadleaf evergreen forest, croplands, built-up, grasslands and bare soils/rocks. Change detection analysis was then carried out for the catchment and sub-catchments in ENVI 5.1 for three periods, 1989–1999, 1999–2009 and 2009–2019. These were used to estimate the probability of increased agricultural intensification in the catchment.

Assessment of agricultural intensification was estimated using an Agricultural Expansion (AE) probability equation (Eq. 1) which was developed by the authors. The probability equation was developed to predict the probability of a catchment exhibiting intensification characteristics, using the addition rule. The equation was specifically used because it allowed for quantifying uncertainty, based on predictive analysis. The AE probability equation is a nominal based econometric formula that assumes that reduced forest area, increased cropland area, reduction in grassland area, and increased bare soils/rocks are associated with increased agricultural intensification. The equation omits built area, assuming that built area may not significantly contribute to agricultural production and, therefore, limited intensification. However, built area may be a driver of intensification, which is a potential limitation of the equation.

1 $$AE = Heterogeneity + Forest \,cover + Cropland + Grassland + Bare \,soil$$

where AE is a cumulative score (5 = full intensification; 3–4 = semi-intensification; 2 = semi-extensive; 1 = extensive); Heterogeneity is 1(otherwise 0) if the number of land use classes have increased; Forest cover is 1 (otherwise 0) if there is a reduction in proportion of broadleaf evergreen forest; Cropland is 1 (otherwise 0) if the there is an increase in the proportion of cropland; Grassland is 1 (otherwise 0) if there is an increase in proportion of grassland; and Bare soil is 1 (otherwise 0) if there is a reduction in proportion of bare soil.

### Determination of nutrients and pesticides emissions in the catchment

The distribution of the TSS, nutrient and pesticide concentration data was tested using a Shapiro–Wilk test and checked visually using Q–Q plots. As the data were not normally distributed they were log-transformed. A Multivariate Analysis of Variance (MANOVA) was used to test differences in concentrations among sampling sites. Linear regression was used to test for the relationship between nutrient and pesticide concentrations across sampling sites. Concentrations among sites were compared using the Kruskal–Wallis Test.

The consequences of nutrient and pesticide concentrations to the aquatic ecosystem were assessed using ratios of likely ecological importance of TN:TP; DDD/DDE, (DDE + DDD):DDT and DDT:DDE; and α:γ-HCH. The stoichiometric TN/TP ratio based on the Redfield ratio^34^ has been used as an indicator of potential N or P limitation for phytoplankton in lakes, with a molar ratio above 16 indicating potential P limitation^35^. The ratio of DDD/DDE is an indicator of the conditions for DDT biodegradation with a ratio > 1 indicating anaerobic DDT biodegradation, whereas a ratio < 1 indicates an aerobic DDT degradation^36,37^. The (DDE + DDD)/DDT ratio is an index for source of DDT, with a ratio > 0.5 associated with long term weathering of DDT from soils^38^. High ratios of DDT:DDE indicate more recent exposure than lower ratios^39,40^. The α:γ-HCH ratio is an indicator of whether technical HCH (low insecticidal properties) or lindane (high insecticidal properties) is being used, with values more than one indicating technical HCH, and values below one indicating lindane use^41,42^.

The estimates of nutrient emissions was augmented by hydrological discharge for the sites. The discharge (*Q* in m^3^/s) was measured using the area-velocity method following Gore and Banning^43^ as a product of the average velocity (V m/s) and the cross-sectional area (*XA* in m^2^) at a site. For daily discharge (*Q*_*e*_ in m^3^ day^−1^) for the sites, daily flow measurements, for the same sampling period as the nutrients and pesticide concentrations, were acquired from the L. Naivasha Water Resources Authority (WRA) for three discharge measuring stations in R. Malewa Highway bridge, Wanjohi, and Gilgil highway bridge. Using regression analysis between the measured discharge and the flow measurements from the WRA, the daily discharge for the sampling period was estimated using Eq. (2):

2 $$Q_{e} = c + m \times Q_{dm}$$

where *Q*_*e*_ is the estimated daily discharge; *c* is the regression intercept; *m* is the regression coefficient; and *Q*_*dm*_ is the daily flow measurement from the current study.

### Relationship between land uses, nutrients and pesticide concentrations

Using mixed model analysis, the study assessed the relationship between land cover categories and the concentration of total phosphorus, total nitrogen, ∑DDT, ∑HCH, and ∑Cyclodienes found in the water samples. The *lme4* function^44^ in R was used to perform a linear mixed effect analysis of the relationship between land use categories and concentrations of the nutrients and pesticides. Linear mixed models with random slopes and intercepts were reduced to the minimum adequate models using the step (model x, direction = “both”) function to remove autocorrelations. The *anova* function in R^45^ was used to analyse for significant differences between complete models of pesticides or nutrients concentrations to determine sensitivity of the models. The random intercept model had lower Akaike’s Information Criterion (AIC) compared with the random slopes was adopted for relating fixed and random effects. The relationship between the concentrations of pesticides and nutrients, and the level of agricultural intensification was estimated using multinomial logistic regression. The multinomial logistic regression was suitable considering that the outcome variable (level of intensification determined by Eq. 1) was nominal, as such, the relationship between land uses and emissions was modeled as a linear combination of predictor variables (concentration of nutrients and pesticides at a site).

### Determining the risks associated with combined emission of nutrients and pesticides

To determine the risks of emissions from combined nutrients and pesticides, a water quality metric was calculated (Eq. 3) based on the ratio between measured in situ concentrations and published water quality standards for the preservation of aquatic life used by (a) the United States Environmental Protection Agency (USEPA) guidelines reported for minimum effect concentrations^46^, and (b) the Kenyan Water Services Regulatory Board (WASREB) Guidelines on Drinking Water Quality And Effluent Monitoring^47^ (Supplementary Table 3).

3 $$Risk_{i} = \frac{{C_{i} }}{{S_{i} }}$$

where *Risk*_*i*_ is probability of effect (Risk) for each of the contaminants (i); *C*_*i*_ is the in-situ concentration of contaminant *i*; and *S*_*i*_ is the water quality standard for the protection of aquatic life.

An evaluation was made separately for total nitrogen (TN) and total phosphorus (TP), where guideline values from both USEPA and WASREB were available. The pesticide guideline values were mainly from USEPA, and not from both USAPA and WASREB.

A water quality risk map was constructed using the Log_10_ of the product of risk ratios determined for each contaminant (Eq. 4). A product less than 1 indicated lower combined risk, while a product greater than 1 indicates higher negative risk potential (Table 1). This equation was developed by the authors to be able to translate the risk ratios to potential of impact of combined nutrients and pesticides. To develop the maps, classified raster images were converted depicting water quality status into vector data with distinct polygons representing different water quality categories. Easting and northing coordinates were collected for sample location. This data was then exported to a spreadsheet in CSV format. Using ArcGIS X, and Y data import tools, the data was transformed into a shapefile. The entire dataset was projected to Arc 1960 UTM Zone 37S and produced the map using ArcGIS software 10.8.2.

4 $$Risk\, calculation = Log_{10} (DDT_{r} \times HCH_{r} \times Cyclo_{r} \times nitrogen_{r} \times phosphorus_{r} )$$

where Risk calculation is classified as in Table 1, *DDT*_***r***_ is the site risk ratio for DDT; *HCH*_***r***_ is the site risk ratio for HCH; *Cyclo*_***r***_ is the site risk ratio for cyclodienes, *nitrogen*_***r***_ is the site risk ratio for nitrogen; and *phosphorus*_***r***_ is the site risk ratio for phosphorus).

**Table 1:** Classification of water quality risk map based on instantaneous in-situ results of the study.

Risk calculation range	Risk description	Risk classification
Less than or equal to 0	The ratios indicate the water quality status is within the standards for protection of water quality	Low ecological risk
Between 0 and 1	The contamination is between one and ten times higher than the standards for protection of water quality	Medium ecological risk
Between 1 and 2	The contamination is between ten and 100 times higher than the standards for protection of water quality	Medium ecological risk
Between 2 and 3	The contamination is between 100 and 1000 times higher than the standards for protection of water quality	Medium–High ecological risk
Between 3 and 4	The contamination is between 1000 and 10,000 times higher than the standards for protection of water quality	High ecological risk
Between 4 and 5	The contamination is between 10,000 and 100,000 times higher than the standards for protection of water quality	High–Very high ecological risk
Above 5	The contamination is above 100,000 higher than the standards for protection of water quality	Very high ecological risk

## Results

### Agricultural intensification in Lake Naivasha catchment

**Figure 2:**
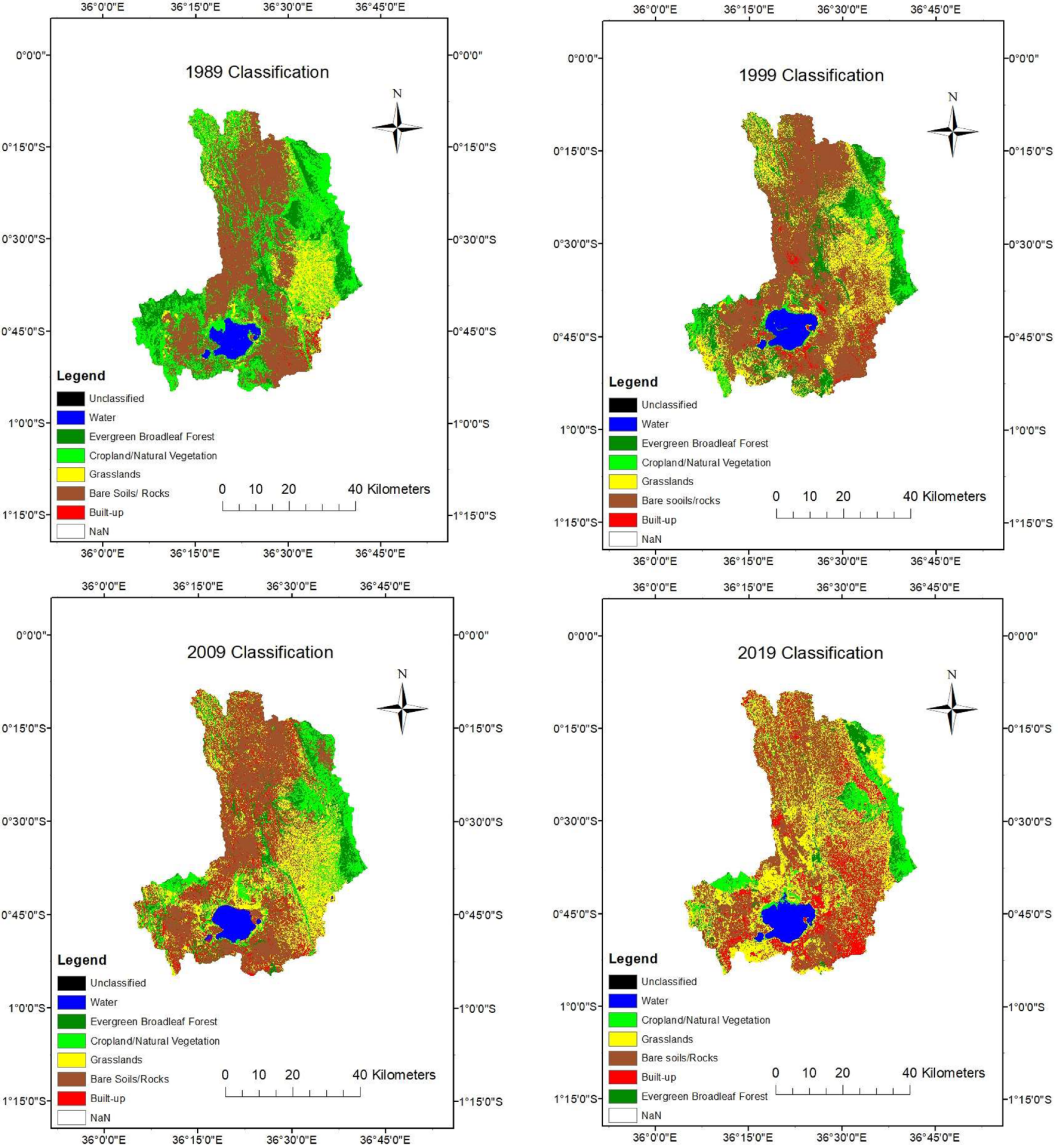
Decadal landuse changes from 1989 to 2019.

In the L. Naivasha catchment, between 1989 and 2019, there was an estimated increase of cropland by 623 km^2^, a reduction of forest cover by 200 km^2^, increase of grasslands by 534 km^2^, a reduction in bare soils by 100 km^2^, and an increase of built area by of 540 km^2^ (Fig. 2; Supplementary Table 4).

The MANOVA results show a significant change in LULC over (Pillai’s Trace = 1.39, *F* (5,40) = 4.28, *p* < 0.05). The ANOVA results indicate significant differences in land use/cover between the sub-catchments of the R. Gilgil and R. Malewa (F (8,199) = 35.52, p < 0.05), and among the different land use classes, especially between bare soils and the other land uses/cover classes (F (5,199) = 19.36, *p* < *0.05*) indicating a reduction of bare soils. There is demonstrated intensified agricultural expansion in the catchment, ranging from potential full intensification at G1, to extensive agriculture in G3 in 2015 relative to 1989 (Supplementary Table 5).

### Nutrients, pesticides and TSS emission in the catchment

The concentrations of nutrients varied from upstream to downstream in the rivers of the catchment (Table 2). The highest total phosphorus (TP) concentration (1551 ± 179 µg L^−1^) was recorded within the R. Karati (K1—Karati highway) and the lowest (42 ± 3 µg L^−1^) within L. Naivasha (N3—Hippo Point). Maximum TP concentrations were recorded in the sequence R. Karati > R. Malewa > R. Gilgil > L. Naivasha, with significant differences among sites (Kruskal–Wallis chi-squared test = 85.30, df = 12, *p* < 0.05), and notably between the rivers and lake sites, and between K1 and the upper reaches of the R. Gilgil (G1—Kahuho, G2—Little Gilgil) and R. Malewa (M1 and M2—upper Malewa. The highest recorded total nitrogen (TN) concentration was found at K1 (2554 ± 574 µg L^−1^) and lowest (521 ± 97 µg L^−1^) at M2. Maximum TN concentrations were recorded in the sequence R. Karati > R. Gilgil > R. Malewa > L. Naivasha, with significant differences among the sites (Kruskal–Wallis chi-square test = 26.64, df = 12, *p* < 0.05), and higher in the river than the lake sites.

**Table 2:** Concentration (mean ± SE) of nutrients and pesticides recorded in L. Naivasha catchment within the study sites (n = 5 monthly samples per site).

Sub-catchment	Site	Nutrient Concentrations		Pesticide residues concentrations		
		TN	TP	∑DDT	∑HCH	∑Cyclodienes
		(µg L^−1^)	(µg L^−1^)	(ng L^−1^)	(ng L^−1^)	(ng L^−1^)
R. Karati	K1 (downstream)	2554 ± 574^H^	1551 ± 179^H^	94 ± 16^L^	135 ± 57	1487 ± 173
R. Gilgil	G1(upstream)	1296 ± 332	140 ± 29	278 ± 73	33 ± 4^L^	1699 ± 489
	G2 (tributary)	835 ± 193	127 ± 19	175 ± 40	187 ± 81	1383 ± 276
	G3 (downstream)	1389 ± 339	244 ± 56	338 ± 73	82 ± 17	256 ± 3^L^
R. Malewa	M1(upstream)	1035 ± 122	138 ± 27	269 ± 44	141 ± 64	989 ± 207
	M2 (upper midstream)	521 ± 97^L^	326 ± 98	157 ± 34	98 ± 21	738 ± 123
	M3 (tributary)	1664 ± 510	594 ± 181	163 ± 39	51 ± 8	618 ± 115
	M4 (mid-stream)	872 ± 167	409 ± 110	168 ± 20	99 ± 22	392 ± 78
	M5 (downstream)	1464 ± 380	502 ± 154	264 ± 82	91 ± 20	1588 ± 404
L. Naivasha	N1	695 ± 158	164 ± 47	294 ± 71	260 ± 102^H^	1762 ± 278
	N2	497 ± 138	51 ± 5	210 ± 65	159 ± 64	1209 ± 66
	N3	597 ± 163	42 ± 3^L^	377 ± 140^H^	238 ± 89	3481 ± 440^H^
	N4	563 ± 153	47 ± 3	352 ± 125	186 ± 68	1916 ± 354

^H^indicates the highest concentration of the contaminant across sites, and ^L^indicates the lowest concentration of the contaminant across sites.

Pesticides recorded comprised the ∑DDT group (p,p-DDT, p,p-DDE and p,p-DDD), ∑HCH group (alpha, beta, delta and gamma), and ∑Cyclodienes group (aldrin and derivative dieldrin; heptachlor and derivative heptachlor epoxide; endrin and its derivative endrin aldehyde; endosulfan group comprising alpha, beta and endosulfan sulphate; and methoxychlor) (Table 2). The concentrations of ∑DDT, ∑HCH, and ∑Cyclodienes varied across the different sub-catchments and sites in the study. However, there was no significant difference among the sites for the pesticide groups (Kruskal–Wallis chi-square test = 12.57, df = 12, *p* < 0.40).

Maximum concentrations of ∑DDT were recorded in the sequence L. Naivasha > R. Gilgil > R. Malewa > R Karati. Among the R. Gilgil sub-catchment, G3 (downstream) showed the highest total ∑DDT concentration, followed by G1 (upstream). Within the R. Malewa sub-catchment, M5 (downstream) had the highest total ∑DDT concentration, while M2 (upper midstream) exhibited the lowest. Among the L. Naivasha sub-catchment, N3 had the highest total ∑DDT concentration, followed by N4. Sites like R. Karati K1 (downstream) and M2 (upper midstream) recorded relatively lower total ∑DDT concentrations.

The concentrations of ∑HCH ranged from 33 ± 4 ng L^−1^ (at G1) to 260 ± 102 ng L^−1^ (at N1), with the maximum concentrations of ∑HCH recorded in the sequence L. Naivasha > R. Karati > R. Gilgil > R. Malewa. The lowest concentration of ∑HCH was recoded from G1 (Kahuho) in the upper part of the catchment. Site N1 in L. Naivasha sub-catchment displayed the highest total ∑HCH concentration. The downstream sites in R. Gilgil and R. Malewa sub-catchments showed varying levels of ∑HCH concentrations, with G3 (downstream) and M5 (downstream) showing higher levels compared to their upstream counterparts.

The ∑Cyclodienes ranged between 256 ± 3 ng L^−1^ at G3 (R. Gilgil Highway Bridge) to 3481 ± 440 ng L^−1^ at N3 (Hippo point). Maximum concentrations of ∑Cyclodienes were recorded in the sequence L. Naivasha > R. Gilgil > R. Malewa > R. Karati. Among the R. Gilgil sub-catchment, G1 (upstream) showed the highest total ∑Cyclodienes concentration, followed by G2 (tributary). Within the R. Malewa sub-catchment, M3 (tributary) had the highest total ∑Cyclodienes concentration, while M4 (mid-stream) exhibited the lowest. Among the L. Naivasha sub-catchment, N3 had the highest total ∑Cyclodienes concentration, followed by N4. Site N1, located within L. Naivasha, also showed a relatively high total ∑Cyclodienes concentration. Other sites like R. Karati K1 (downstream) and G3 (downstream) recorded comparatively lower total ∑Cyclodienes concentrations.

In reference to the relationship between nutrients and pesticides concentration, it was established that the concentrations of nutrients (total phosphorus and nitrogen) as independent variables were not significant predictors of the concentrations of the majority of pesticides (∑HCH and ∑Cyclodienes) in the catchment (Table 3). While TN had a significant relationship with concentration of ∑DDT (F(1,89) = 4.28, *p* < 0.05, R^2^ = 4%), where an increase of TN predicted a reduction of ∑DDT, however, the very low R^2^ indicates very weak predictive power.

**Table 3:** Linear regression results between the concentration of nutrients and pesticides in the L. Naivasha catchment.

Pesticide residues	Total nitrogen		Total phosphorus	
	Coefficient^a^	Statistics	Coefficient^a^	Statistics
∑DDT	− 0.04 ± 0.02*	F(1,89) = 4.28, *p* < 0.05, R^2^ = 4%	− 0.07 ± 0.04	F(1,86) = 2.13, *p* = 0.15, R^2^ = 1%
∑HCH	0.02 ± 0.01	F(1,121) = 3.50, *p* = 0.06, R^2^ = 2%	− 0.04 ± 0.04	F(1,118) = 1.27, *p* = 0.26, R^2^ = 0%
∑Cyclodienes	− 0.08 ± 0.19	F(1,46) = 0.17, *p* = 0.68, R^2^ = 0%	− 0.44 ± 0.34	F(1,45) = 1.74, *p* = 0.19, R^2^ = 1%

^a^Coefficient ± Standard Error.

*Statistically significant.

**Figure 3:**
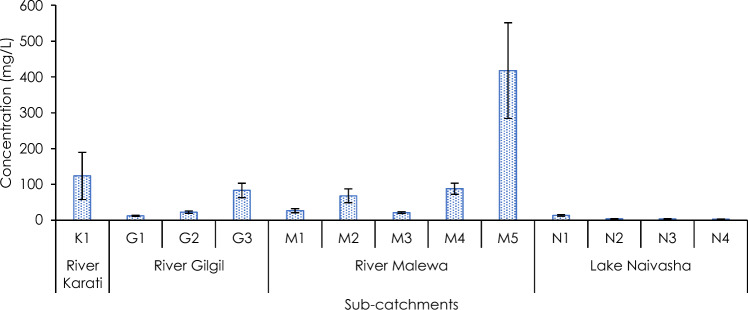
Concentration (mean ± SE) of total suspended solids (TSS) in the sub-catchments (n = 5 samples per site). Error bars indicate the Standard Error of mean (SE).

The highest concentration (418 ± 133 mgL^−1^) of total suspended solids (TSS) was recorded in the R. Malewa (at M5), and lowest in the lake, ranging between 2.2 ± 0.8 and 13.5 ± 2.2 mgL^−1^ (Fig. 3). Within the R. Malewa and R. Gilgil, the concentration of suspended solids increased from upstream (M1 and G1, respectively) to downstream (M5 and G3, respectively). Concentrations of suspended solids in R. Karati were higher than in R. Gilgil (ranging: 12.2 ± 1.4 mgL^−1^ and 82.9 ± 19.8 mgL^−1^), and lower than R. Malewa (range: 21.1 ± 2.4 mgL^−1^ and 418 ± 133 mgL^−1^). There was a significant difference among the sub-catchments on the suspended solids (Kruskal–Wallis chi-squared = 86.79, df = 12, *p* < 0.05), especially between the river sub-catchments and the lake sites (Kruskal–Wallis chi-squared = 62.24, df = 3, *p* < 0.05).

A simple regression analysis was performed to estimate the strength of TSS as a predictor of nutrients and pesticides emissions. There was no significant prediction probability of TSS on the emissions, with R^2^ of between 1 and 9% for the pesticides, and 14–15% for the nutrients (Table 4).

**Table 4:** Linear regression results between total suspended solids and the concentration of nutrients and pesticides in the L. Naivasha catchment (*df* = 1,12).

Nutrients/Pesticides	F	*p* value	R^2^
TP	1.853	0.2007	0.144
TN	2.018	0.1832	0.155
∑DDT	0.150	0.7060	0.013
∑HCH	1.121	0.3123	0.093
∑Cyclodienes	0.094	0.7647	0.008

Nutrients and pesticides ratios are used as inference of the emissions (Table 5). The TN:TP ratios were only considered for the lake sites considering that the Redfield ratio was developed for lentic systems^48^, and applying the ratio to lotic systems brings in a number of additional complexities^34^. Except for N3 where the molar ratio between TN and TP was above 16 indicating a potential phosphorus limitation, for other sites molar TN:TP ratio was lower than 16. Although the stochiometric ratio indicated P-limitation, the measured concentration of TP was relatively high at N3 (42 ± 3 µg L^−1^), indicating low probability that phosphorus (if in available form) was limiting for biological production.

**Table 5:** Ratios for pesticides and nutrients in L. Naivasha catchment.

Site	DDD:DDE	(DDE + DDD):DDT*	DDT:DDE**	TN:TP	α:γ-HCH
K1	0.58	0.1	11.07	2	0.8^c^
G1	1.75^b^	0.1	19.51	21^a^	0.76^c^
G2	1.88^b^	0.2	15.72	17	0.49^c^
G3	0.87	0.5	4.02	11	0.71^c^
M1	2.49	0.3	12.45	11	1.43^d^
M2	0.55	0.2	6.9	5	1.02^d^
M3	0.95	0.2	8.03	7	0.77^c^
M4	1.27^b^	0.2	13.95	12	1.15^d^
M5	1.91^b^	0.3	8.86	6	0.28^c^
N1	0.94	0.3	6.42	6	0.24^c^
N2	0.78	0.2	8.58	11	0.18^c^
N3	0.63	0.2	6.82	18^a^	0.32^c^
N4	2.27	0.5	7.17	11	0.17^c^

*Values indicate source of DDT is not from long term weathering (see section “Determination of nutrients and pesticides emissions in the catchment” for explanation regarding ratios).

**Values indicate recent DDT applications (see section “Determination of nutrients and pesticides emissions in the catchment” for explanation regarding ratios).

^a^Potential P-Limitation.

^b^Areas of anaerobic DDT degradation.

^c^Use of lindane with higher insecticidal effect.

^d^Use of technical HCH with lower insecticidal effects.

The DDD:DDE ratio was highest in the upper reaches of R. Gilgil (1.75–1.88) and the lower reaches of the R. Malewa (1.27–1.91). In the L. Naivasha sites, the DDD:DDE ratio was the lowest (ratio range). This suggests that DDT degradation was anaerobic in the rivers (sediments in the pools) and aerobic (pelagic) in the lake (see section “Determination of nutrients and pesticides emissions in the catchment” for explanation regarding inference of ratios). The ratio of (DDE + DDD):DDT recorded ranged between 0.1 and 0.5, an indication that the DDT found in the water is not from long term weathering of soils. Further, the DDT:DDE ratio recorded at all the sites were above 0.33, indicating recent use of pesticides with DDT as active ingredients. The α:γ-HCH ratios were more than one in the upper and mid reaches of the R. Malewa, an indication of the use technical HCH with lower insecticidal potential, while in the R. Gilgil and the lake sites showed ratios of below one, indicating use of lindane, with higher insecticidal effects. The L. Naivasha sites showed the lowest α:γ-HCH ratios suggesting higher use of lindane in the catchment of the lake.

In this study, the lowest hydrological discharge was recorded at Little Gilgil (G2), and the highest at Malewa Highway Bridge (M5) (Table 6).

**Table 6:** Mean daily discharge measurements at the L. Naivasha catchment sampling sites, for the sampling period (n = 5).

Sub-catchment	Site		Mean daily discharge ± S.D. (m^3^ s^−1^)
R. Gilgil	G1	Located in the Upper reaches of the R. Gilgil catchment	0.35 ± 0.51
	G2	On tributary to the main stem, in the mid-reaches of the sub-catchment	0.03 ± 0.01
	G3	Site in the lower reaches of the catchment below the Gilgil Dam, along the main highway	0.74 ± 0.67
R. Karati	K1	Site along the R. Karati– the Karati Highway Bridge site (K1), is in the lower reaches of the river, along the main highway	0.39 ± 0.55
R. Malewa	M1	Located in the upper reaches of the sub-catchment	0.11 ± 0.04
	M2	Site at the upper reaches of the sub-catchment	0.45 ± 0.27
	M3	On a tributary to the main stem, in the mid reaches of the sub-catchment	1.20 ± 1.31
	M4	Site in the mid-reaches of the sub-catchment, below the Turasha Dam	1.81 ± 1.05
	M5	Located in the lower reaches of the sub-catchment, along the main highway	2.95 ± 1.78

### Relationship between intensification and emission of nutrients and pesticides

In the study, the potential effect of LULC changes indicate various potential scenarios. For example, increasing forest cover could potentially reduce contamination from nutrients (TP and TN), while increasing pesticide residues (∑DDT, ∑HCH and ∑Cyclodienes) within the surface waters of the L. Naivasha catchment (Table 7). The findings indicate a reduction in MCNV, EGBF, and SGL predicted the increase in emission of TP, while a reduction in MCNV, EGBF and BSBA would predict an increase in TN. LULC types were less likely to predict the emission of pesticides, except for cyclodienes where an increase of all the four LULC types predicted an increase of ∑Cyclodienes emission. The relationship between agricultural expansion and concentration of nutrients and pesticides indicated differing influences of land use and intensification on pesticide and nutrient concentrations (more detail in Supplementary Table 6). For instance, the findings indicate that an ecosystem with semi natural habitats would have lower concentrations of TP, and ∑DDT, and a higher concentration of TN compared with a polluted ecosystem.

**Table 7:** Potential effects of land use/cover on the concentration of nutrients and pesticides.

Model variables	Fixed effects estimate ± Standard error				
	Total phosphorus (µg L^−1^)	Total nitrogen (µg L^−1^)	∑DDT (ng L^−1^)	∑HCH (ng L^−1^)	∑Cyclodienes (ng L^−1^)
Intercept	5.6 ± 0.72	7.56 ± 1.81	1.16 ± 0.67	1.96 ± 0.15	− 33.61 ± 12.01
MCNV	− 4.13 ± 0.85***	− 4.47 ± 1.67**	2.10 ± 1.32	− 0.67 ± 0.25**	40.92 ± 13.43**
EGBF	− 71.77 ± 10.64***	− 41.84 ± 12.52***	39.89 ± 18.43*		77.94 ± 26.92**
SGL	− 9.93 ± 2.44***	− 1.58 ± 2.96	7.06 ± 3.73		23.12 ± 8.36**
BSBA		− 5.86 ± 2.39*			45.13 ± 15.60**

*MCNV* mixed cropland and natural vegetation, *EGBF* evergreen broadleaf forest, *SGL* shrubland/grasslands, *BSBA* bare soils/build area.

*< 0.05 level of significance.

**< 0.01 level of significance.

***< 0.001 level of significance in the prediction.

### Potential effects of emission to aquatic ecosystems

For all except the Karati Highway site (K1), the concentrations of ∑DDT and ∑Cyclodienes found in the surface waters of the L. Naivasha catchment exceed 100 times the water quality standards recommended by US EPA^46^ (more detail in Supplementary Table 2). Other than K1, TN concentrations were modest across all sites according to the US EPA standards^46^, and lower than those provided for Kenya^47^. However, TP concentrations ranged from high to extremely high for river waters, although the concentrations were below water quality standard concentrations for protection of aquatic life indicated by both USEPA and WASREB standards. While the risk of pesticides (∑DDT and ∑Cyclodienes) to aquatic life was lower in K1, the site had high concentrations of TP. In general, the lake showed the lowest risk ratios, while R. Malewa catchment showed the highest risk ratios.

**Figure 4:**
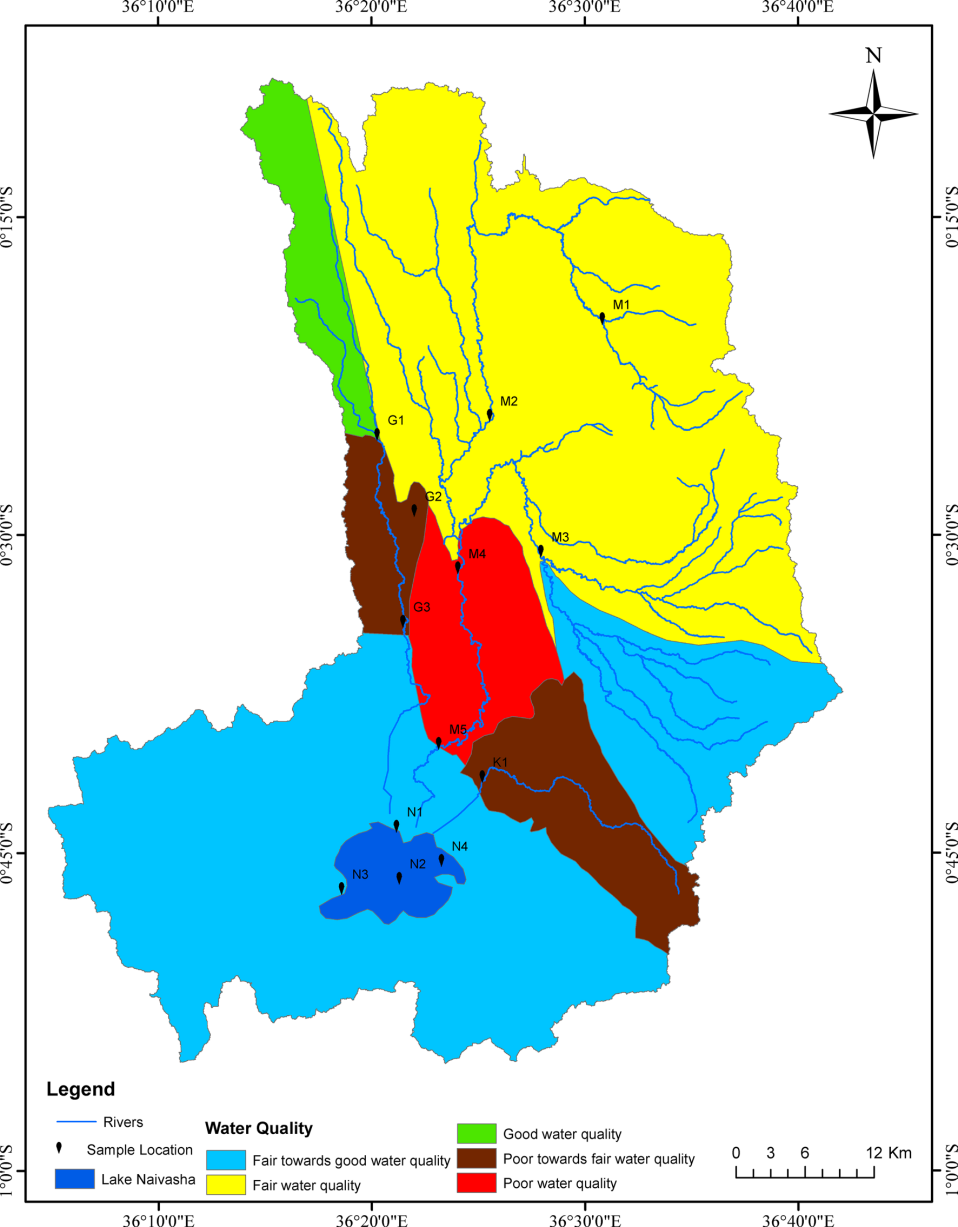
Water quality risk map for the sub-catchments of the Lake Naivasha catchment, Kenya. Sampling sites are: G1 = Kahuho, G2 = Little Gilgil, G3 = Gilgil Highway Bridge, M1 = Wanjohi, M2 = upper Malewa, M3 = Turasha, M4 = Bush Ventures, M5 = Malewa Highway Bridge, K1 = Karati Highway Bridge, N1 = River Mouth, N2 = Mid-lake, N3 = Hippo Point, N4 = Crescent.

The water quality risk map (Fig. 4) based on criteria shown in Table 1, indicates that most of the lower reaches of the rivers (R. Gilgil, R. Malewa and R. Karati), tend towards poor water quality for the five pollutants (TN, TP, ∑DDT, ∑HCH and ∑Cyclodienes) recorded in the study. Specifically, the upper reaches of R. Gilgil (at G1), showed good water quality, while the upper reaches of R. Malewa had fair water quality status. While the lower reaches of R. Karati (at K1) and R. Gilgil (at G3) showed poor to fair water quality, the lower reaches of R. Malewa (at M5) showed poor water quality.

## Discussion

### Agricultural intensification in Lake Naivasha catchment

In the 20 years between 1989 and 2019, the extent of grassland in the lower part of the L. Naivasha catchment, and cropland and human population in the upper part has increased^30,49^. The catchment has diverse agriculture comprising crop cultivation, livestock rearing, and horticulture. This has implications for the potential export of both nutrients and pesticides from land to water, and consequences for the ecosystem health of the rivers and lake. Increased population density in the upper catchment to above 600 persons per km^2^ is associated with smaller division of land holdings, and the need for greater application of nutrients and pesticides^3^. Smallholder farmers in the L. Naivasha catchment grow a variety of crops including maize, beans, wheat, and vegetables, while cattle, sheep, and goats are reared for both commercial and subsistence livelihoods. Additionally, Sulastri et al.^50^ reports a thriving horticulture in the 50 km^2^ around the lake, especially floriculture, which increases the potential application of N and P fertilisers^50^. While the model used in this study indicates that extensive agricultural systems had a higher probability to export nutrients and pesticides than intensive agricultural systems, this also depends on the nature of the land management, reflected in the spatial pattern of nutrients and pesticides detected from field sampling.

### Nutrients and pesticides emission in the catchment

Maximum nutrient concentrations in the catchment found at K1 (R. Karati Highway Bridge) could be associated with the number of farms close by, that likely provide point sources from farm drains. Mutia et al.^51^ and Ogendi et al.^52^ have both shown the Karati highway bridge site (K1) to have high concentrations of heavy metals, as well as nutrients as also shown in this study. Although Everard et al.^53^ postulated that the geological difference between the R. Karati sub-catchment and the other river sub-catchments could explain the high chemical contamination at K1, the high concentrations of TP (1551 ± 179 ug TP l^−1^) found at the site suggest point-source pollution. According to a USGS report, TN concentrations in rivers and streams in the United States range from less than 0.1 to more than 100 mgL^−1^, with a median value of approximately 1.5 mgL^−1^. Median TP concentrations in US streams range from approximately 0.01 to 3.3 mgL^−1^^54^. On the other hand, according to the European Environment Agency (EEA), median TN concentrations in rivers in Europe range from less than 0.1 to approximately 5 mgL^−1^, with the highest concentrations observed in southern Europe. Median TP concentrations range from approximately 0.02 to 0.3 mgL^−1^^55^. Comparatively, our study show that the recorded concentrations are within the ranges of the United States and the European rivers.

The high discharges in R. Malewa (upto 2.95 m^3^ s^−1^) compared with the other rivers (highest 0.74 m^3^ s^−1^ in R. Gilgil, and 0.39 m^3^ s^−1^ in Karati) in the L. Naivasha catchment results in high loads (up to 4.32 g s^−1^ of TN, and 1.48 g s^−1^ of TP) of nutrients from the R. Malewa catchment, compared with 1.03 g s^−1^ TN and 0.18 g s^−1^ TP in R. Gilgil, and 0.996 g s^−1^ TN and 0.60 g s^−1^ TP in R. Karati. However, the large area of the R. Malewa catchment, and the relatively lower nutrients concentrations compared with R. Karati, makes R. Karati a significant contributor to nutrients yield (of 1 g ha^-1^ s^-1^) into the lake per unit area. The R. Karati had the highest concentrations of total nitrogen (TN) of the rivers in the L. Naivasha catchment. The lake itself had the lowest TN concentrations, as expected and commonly recorded in many lakes^56–58^. The lower concentrations in receiving water bodies have been related to sedimentation^58^ in lakes and bio-uptake for instance by the biofiltration of the north swamp of L. Naivasha, or dilution^57^ across inputs.

Yongo et al.^59^ attributed the transition of L. Naivasha from eutrophic to hypereutrophic status to increased nutrient inputs. The transition of, especially TP, between the lower reaches of the influent rivers, the river mouth and the open lake suggests settlement in the lake predicted by traditional loading models^58^. It is also apparent that compared with measurements made in 2002 by Kitaka et al.^60^, concentrations of P in the lake have increased approximately three-fold. In this study we report the TN:TP ratios in the rivers, although interpreting TN:TP ratios and P limitation in rivers can be problematic^61,62^.

Despite a ban on DDT use except by public health officials^22,63^, for the control of vector-borne diseases similar to South Asian countries—particularly India^64^, the pesticide results suggest its continued use in the L. Naivasha catchment. This study recorded a 100-fold higher concentration compared with the US EPA water quality standards. That the ∑DDT was highest in the lake samples suggests accumulation and DDT resuspension due to bioturbation and wave action that can re-release DDT into the water column^65^. Moreover, the long environmental half-life of DDT^66^ could mean that DDT flushed from the rivers, is trapped within the lake at higher concentrations^67^. However, high DDT:DDE ratios across the catchment indicate recent and high inputs in the catchment. A global review by Vasseghian et al.^68^ identifies higher concentrations of p,p′-DDT compared with other persistent organic pollutants, aligning with the findings from this study of ∑DDT having highest concentrations. The elevated levels of ∑DDT in certain areas, such as G3 (downstream) in the R. Gilgil sub-catchment, M5 (downstream) in the R. Malewa sub-catchment, and N3 in the L. Naivasha sub-catchment, indicate a concerning presence of persistent organic pollutants in the catchment. ∑DDT, known for its persistence and bioaccumulative properties, poses long-term risks to aquatic ecosystems and human health^39,69^. The identification of these hotspots is crucial for prioritizing remediation efforts and implementing measures to reduce the input of DDT compounds into the catchment. Comparatively, the current study recorded concentrations higher than recorded (1.6 to 170 ngL^−1^) in China^70^, and lower (< 1 ngL^−1^ to 1500 ngL^−1^) than recorded in India^71^.

That ∑HCH was highest in the lake suggests continued application of technical lindane around the lake as reported by Onyango et al.^22,72^, but up to seven times the values recorded in 2011 by Njogu^73^. The spatial distribution of ∑HCH, with site N1 exhibiting the highest concentration within the L. Naivasha sub-catchment, and elevated levels observed in downstream areas of R. Gilgil and R. Malewa sub-catchments, highlights the need for targeted monitoring and remediation strategies^74^. The presence of higher ∑HCH levels in downstream sites suggests potential accumulation and transport processes, emphasizing the interconnected nature of water systems within the catchment and the importance of addressing pollution sources upstream to prevent downstream impacts^75^. The concentrations recorded (33 to 260 ngL^−1^) in this study are lower compared with the levels (3.9 to 87 ngL^−1^) in China^70^, and lower (< 1 ngL^−1^ to 700 ngL^−1^) than those recorded in India^71^, an indication that the pesticide pollution in L. Naivasha is lower than in India, although both countries use the pesticides for vector diseases control.

The concentrations of ∑Cyclodienes were highest in the lake sites, the Gilgil river, and lower reaches of R. Malewa. The values recorded in this study were higher compared with the concentrations recorded in 2002 by Gitahi et al.^76^, suggesting continuous increase in use of cyclodienes based pesticides. The distribution of the cyclodienes within the lake showed high concentrations within the deeper parts of the lake with relatively lower anthropogenic disturbance (M3—Hippo point), as documented by Ndungu et al.^27^ and Outa et al.^74^. The variations in ∑Cyclodienes concentrations across the different sub-catchments reflect unique contamination patterns within each area. The higher levels observed in certain tributaries and downstream sites signify potential localized sources of Cyclodiene pesticides^77^, indicating the influence of specific land uses, agricultural practices, or point source inputs. Understanding these sources is crucial for designing tailored interventions to mitigate pesticide inputs and protect water quality.

### Relationship between intensification and nutrients and pesticides emission

Agricultural intensification can have profound effects on aquatic ecosystems, leading to significant changes in water quality, biodiversity, and overall ecological balance. In this study, we show that agricultural intensification in L. Naivasha catchment contributes to nutrient enrichment in the rivers and eutrophication in the lake. Having recorded higher nutrients and pesticides concentrations compared with previous studies such as Kaoga et al.^78^ with data from 2010, Otieno et al.^79^ with data from 2011, and Onyango et al.^22^ with data in 2012, coupled with increased land use on cropland, the potential of excessive nutrient and pesticides runoff or leaching from agricultural fields into nearby water bodies is inevitable. In their study on the relationship between wetlands and nutrients-based pollution in Ontario, Canada, Stempvoort et al.^80^ found a significant positive correlation between nitrogen and land uses, presenting a reverse of the findings illustrated in this study. This emphasizes the potential that depending on the main land uses in a catchment, the relationship between intensification and fertilizer-based nutrients would vary. Notably, that the study by Stempvoort et al.^80^, focuses on a geographic location with well-defined seasons of low and high productivity, while the L. Naivasha catchment is considered productive all through the year. Nevertheless, the study by Giri^81^ related higher potential of pesticides emission from greenhouses growing roses in Kenya, to degraded water quality, emphasizing the emission potential from agricultural related land uses into water bodies.

High pesticide residues associated with forest areas are comparable with the concentration of DDT reported by Onyango et al.^22^ within the upper reaches of the R. Malewa sub-catchment. This is somewhat counter-intuitive, but could reflect that forested areas in the L. Naivasha catchment are associated with fragmented, and small scale, intensified agriculture involving pesticide use for crops such as maize, beans, wheat, and vegetables which are prone to insect and fungal attacks in the area. While this study argues that pesticides and nutrients are used together in intensification, the study found no significant relationship between pesticide and nutrient concentrations. Both the temporal intensity of sampling and differences in modes of action between nutrients and pesticides^82,83^ and the pathways of transfer to aquatic sources^84,85^ could account for this.

### Potential impact of agricultural intensification

Agricultural intensification has undeniable importance for meeting global food demand, but it also poses significant impact on aquatic ecosystems. The impacts of intensified agriculture on water bodies are multifaceted, encompassing nutrient enrichment, pesticide contamination, sedimentation, hydrological alterations, and biodiversity loss^86^. Addressing these impacts requires a comprehensive and integrated approach that combines sustainable agricultural practices, land use management, and effective monitoring and regulation^87,88^. Only through such measures can the adverse effects of agricultural intensification on aquatic ecosystems be mitigated, ensuring the long-term health and sustainability of water resources and the biodiversity they support.

This study applied a risk-based approach to guide better catchment management. Notably the approach involved identifying and assessing potential risks to the L. Naivasha ecosystem and prioritizing management actions based on the level of risk posed. However, as a result nutrients and pesticides emission related stressors, the study identifies the catchment to be of fair to poor water quality state. Notably, the approach applied did not consider other stressors and threats to the catchment, such as habitat degradation, and water abstraction. Nonetheless, the study has identified needs for more focussed assessment of the nutrient and pesticide combined risk to water quality. As a basis, the study has mapped out the lower reaches of the catchment to require more management attention, as there is cumulative contamination from upstream, with higher volumes of flow, amidst increased agricultural activities, especially from floriculture. The documented LULC fragmentation is poised to continue, on one hand because of population increase demanding land fragmentation and on another, the pressure to grow food for local consumption and export outside of the catchment. As a management tool, a risk map presents catchment managers with an entry point on water quality management, and a guide for monitoring. This allows for the allocation of limited resources and implementation of targeted measures to areas of highest risk, maximizing the efficiency and effectiveness of management efforts. Additionally, a risk-based approach promotes adaptive management, as ongoing monitoring and evaluation help to refine strategies and address emerging risks. By adopting a risk-based approach, river catchment management can proactively address threats, protect water quality, preserve biodiversity, and ensure the sustainable use of water resources for present and future generations^89,90^.

The study identified recent and continued emission of pollutants from agriculture to the surface waters of the L. Naivasha catchment. This includes potential impact from banned pesticides used across the catchment, land use changes with complementary intensification practices, and potentially high to very high risk of combined nutrients and pesticide chemical pollution of surface waters. These risks occur across many agricultural catchments in sub-Sahara Africa (SSA)^91–94^. Standards for managing aquatic resources are highlighted in the Africa Water Vision 2025^95^ advocating for a revision of water regulations and laws to give attention to water quality management. The findings in this study illustrate the importance for an Africa Water Vision 2025 that incorporates and attends to the management of combined nutrients and pesticide use and their emissions from land to water.

#### Management of intensification mediated emissions

It is clear that there is a need for long-term monitoring and integration of multiple data sources to better quantify and understand the movement of nutrient from the catchment to the lake^96,97^. In comparing these nutrient concentrations with global catchments, it is essential to consider land uses, since they influence nutrients emissions. Agricultural intensification and land cover have markedly increased nutrient loads into and fluxes within aquatic environments at both global and regional scales^98,99^. The increasing nutrient concentrations observed in the Lake Naivasha catchmentreflect trends seen in other catchments undergoing agricultural development^98,99^such as in Ghana^100^, India^101^ and Canada^80^.

Kenya’s regulations on water quality standards^102^ does not consider the potential of combined effects of nutrients and pesticide, neither does it consider monitoring, review, and policing framework for banned substances in surface waters. Moreover, the standards do not have provisions to monitor effluents associated with banned pesticides. This is a clear indication of a mismatch between regulation and enforcement, that requires the responsible agencies such as the Kenyan Water Resources Authority (WRA), National Environment Management Authority (NEMA), WASREB, and the Kenya Bureau of Standards (KEBS) to actively revise the water quality standards, enforce the management of the standards, and invest in policy and institutional reforms to address management of agricultural intensification.

Policy and institutional reforms contributing to development of regulations that consider the increased risks to surface water and promotes enforcement for compliance to the regulations can provide considerable benefits for sub-Saharan Africa agriculture. Further, the partnerships among national and subnational governments and the private sector can promote coherence of regulations across jurisdictions, and coordination among strategic water quality management and policy authorities. Catchment management partnerships—such as Imarisha in Naivasha^103^,—as a self-organized community for water resources management^104^, have an opportunity for inclusive and integrated management. At the same time, a monitoring regime such as the one proposed in the L. Naivasha Basin Integrated Management Plan for 2012–2022^105^, still require availability of consistent and continuous water quality data, to track and document progress in the development of water resources regulations and policy^106^. The water quality guidelines in Kenya provides for frequency of monitoring, single chemical and biological standards, methodologies for collection and analysis of samples, and a reporting framework^47^. However, the standards do not take into consideration combined contamination. Moreover, strategies developed are still seen to enhance marginalization of local stakeholders, reducing the potential for ownership, and therefore enforcement of any pollution reduction measures^107^. The possible solution to bridging the gap of monitoring combined nutrients and pesticides emissions, with agricultural intensification and land use changes may require continuous biotic monitoring of lakes and rivers in addition to water chemistry. Their presence, abundance, and diversity can serve as indicators of ecosystem health and water quality conditions. Numerous studies have demonstrated the effectiveness of macroinvertebrates as bioindicators in assessing water quality and ecological integrity^108–111^. The use of metrics derived from macroinvertebrate communities such as SASS^112^, TARISS^113^ and KISS^114^, for example, allows for the evaluation and comparison of water quality across different sites and over time^115^. These approaches provide a rapid and cost-effective biological criterion for assessing and monitoring the condition of aquatic ecosystems, that water resources stakeholders refer to in maintaining the integrity of water resources^116^. Intensification, accompanied by increased application of nutrients and pesticides^4^, needs sustained advisory capacity in the use of agricultural inputs^117^. However, the capacity and resources to manage the advisory services are still underdeveloped in Kenya, as they are in many sub-Saharan African countries. Inadequate resources to manage advisory services is exacerbated by inadequate standardized monitoring methodologies. The United Nations Environment Programme in its Progress on Ambient Water Quality 2021 update^118^, report findings from methodological considerations for monitoring water quality. However, the report recommends that the monitoring of ambient water quality should use national and/or subnational water quality standards. In many SSA countries, the standards are not comprehensive, a situation that reinforces the need for integrated review for water pollution management at national level and development of local standards. Liess et al.^119^emphasize need for effective monitoring and management strategies to mitigate impact of persistent pesticides in aquatic systems.

An integrated review of standards would contribute to achieving and compliance with the Sustainable Development Goals (SDGs) including: SDG 2 through promotion of sustainable agriculture practices, SDG 14 through reduction of risks to aquatic biota, SDG 3 through reducing the potential of health complications from exposure to contaminants in the aquatic systems, SDG 6 through availing better water quality and promoting sanitation, and SDG 17 through promoting inclusive partnerships for monitoring and review. Further SDG indicator 6.3.2, provides a mechanism for determining whether, and to which extent, water quality management is successful, with a target to increase the proportion of water bodies with good water quality^118^. The findings from this study, emphasizes the need for water quality managers to consider combined contamination from nutrients and pesticides, and how that is monitored.

## Conclusions

The transformation of the L. Naivasha landscape suggests unsustainable agricultural expansion with fragmented land use/cover, reduced forest cover and grasslands, and increased croplands. This scenario is common in agricultural catchments in sub-Saharan Africa, where forests and grasslands are cleared and replaced by agricultural production of crops and use by livestock. The expansion is linked strongly to increased emissions of nutrients and pesticides to aquatic resources within the catchment, resulting in pollution loads, exceeding legal limits. L. Naivasha catchment is not an exception to this, and this study has demonstrated recent and continued pesticide contamination of highly persistent DDT (and its degradates DDE and DDD). Further, the nutrients enrichment status in the catchment, compared with historical reported enrichment, indicate a catchment that is becoming increasingly eutrophic. These are clear indicators of a catchment with agricultural practices leading to negative combined nutrients and pesticides impact. The catchment exemplifies problems that are widespread across sub-Saharan Africa.

Achieving sustainable catchment management needs the inclusion of combined pollutants as a component for management. Since this is an emerging topic of importance in sub-Saharan Africa, the study highlights the need to adopt practices that support water quality regulators and catchment managers in their ambitions for a more sustainable agriculture, and through that to make progress in achieving the SDGs.

### Supplementary Information

Supplementary Table 1.

Supplementary Table 2.

Supplementary Table 3.

Supplementary Table 4.

Supplementary Table 5.

Supplementary Table 6.

## Data Availability

All data generated or analysed during this study are included in this published article and its supplementary information files.
