# Supplementary material for: Agricultural intensification in Lake Naivasha Catchment in Kenya and associated nutrients and pesticides pollution
Source: Sci Rep. 2024 Aug 9;14:18539. doi: 10.1038/s41598-024-67460-5 (PMC11315982; doi:10.1038/s41598-024-67460-5)
Supplement: Supplementary file 1 — Supplementary Table 1. [file 41598_2024_67460_MOESM1_ESM.docx]

**Supplementary Table 1: CAS numbers and the quality control measures (recovery rates and limits of detection (LOOD)) of the pesticide residues in the study (n=5).**

| **Pesticide** | **CAS Number** | **Recovery  rate (% ± S.D.)** | **LOOD  (µg/L) ± S.D.** |
| --- | --- | --- | --- |
| ***HCH Group*** |  |  |  |
| α HCH | CAS RN: 319-85-6 | 94.82 ± 8.31 | 0.0011 ± 0.0001 |
| β HCH | CAS RN: 319-85-7 | 87.52 ± 4.09 | 0.0016 ± 0.0001 |
| γ HCH | CAS RN: 58-89-9 | 92.06 ± 9.58 | 0.0016 ± 0.0001 |
| δ HCH | CAS RN: 319-86-8 | 82.54 ± 6.95 | 0.0016 ± 0.0001 |
| ***DDT Group*** |  |  |  |
| p,p DDT | CAS RN: 50-29-3 | 91.89 ± 3.41 | 0.0017 ± 0.0001 |
| p,p DDE | CAS RN: 72-55-9 | 78.35 ± 5.12 | 0.0018 ± 0.0001 |
| p,p DDD | CAS RN: 72-54-8 | 79.31 ± 2.84 | 0.0016 ± 0.0001 |
| ***Cyclodienes Group*** |  |  |  |
| α- endosulfan | CAS RN: 115-29-7 | 82.58 ± 4.95 | 0.0011 ± 0.0001 |
| β endosulfan | CAS RN: 33213-65-9 | 93.23 ± 7.13 | 0.0015 ± 0.0001 |
| Endosulfan sulphate | CAS RN: 1031-07-8 | 78.25 ± 10.56 | 0.0021 ± 0.0001 |
| Aldrin | CAS RN: 309-00-2 | 94.26 ± 5.23 | 0.0036 ± 0.0001 |
| Dieldrin | CAS RN: 60-57-1 | 94.83 ± 3.33 | 0.0031 ± 0.0001 |
| Endrin | CAS RN: 72-20-8 | 70.21 ± 4.21 | 0.0022 ± 0.0001 |
| Endrin aldehyde | CAS RN: 7421-93-4 | 77.81 ± 8.63 | 0.0022 ± 0.0001 |
| Heptachlor | CAS RN: 76-44-8 | 92.08 ± 4.56 | 0.0011 ± 0.0001 |
| Heptachlor epoxide | CAS RN: 1024-57-3 | 88.35 ± 2.45 | 0.0011 ± 0.0001 |
| Methoxychlor | CAS RN: 72-43-5 | 88.23 ± 6.86 | 0.0016 ± 0.0001 |
