# Supplementary material for: Agricultural intensification in Lake Naivasha Catchment in Kenya and associated nutrients and pesticides pollution
Source: Sci Rep. 2024 Aug 9;14:18539. doi: 10.1038/s41598-024-67460-5 (PMC11315982; doi:10.1038/s41598-024-67460-5)
Supplement: Supplementary file 2 — Supplementary Table 2. [file 41598_2024_67460_MOESM2_ESM.docx]

**Supplementary Table 2: The potential risk of pesticides and nutrients to aquatic life, relative to the accepted concentrations in the water quality standard**

| **Site** |  | | | | |  |  | Risk ratio calculation | Risk category |
| --- | --- | --- | --- | --- | --- | --- | --- | --- | --- |
|  | ∑DDT (USEPA) | ∑HCH (USEPA) | ∑Cyclodienes (USEPA) | TN (USEPA) | TP (USEPA) | TN (Kenya) | TP (Kenya) |  |  |
| K1 | 62.8 | 0.14 | 63.9 | 2.55 | 23.10 | 1.275 | 11.55 | 4.53 | Very high risk |
| G2 | 117 | 0.20 | 425 | 0.84 | 1.90 | 0.42 | 0.95 | 4.19 | Very high risk |
| G3 | 225 | 0.09 | 346 | 1.39 | 3.64 | 0.695 | 1.82 | 4.53 | Very high risk |
| M1 | 179 | 0.15 | 247 | 1.04 | 2.06 | 0.52 | 1.03 | 4.15 | Very high Risk |
| M2 | 105 | 0.10 | 185 | 0.52 | 4.86 | 0.26 | 2.43 | 3.70 | High risk |
| M3 | 108 | 0.05 | 154 | 1.66 | 8.87 | 0.83 | 4.435 | 4.12 | Very high Risk |
| M4 | 112 | 0.10 | 98 | 0.87 | 6.11 | 0.435 | 3.055 | 3.78 | High risk |
| M5 | 176 | 0.10 | 397 | 1.46 | 7.49 | 0.73 | 3.745 | 4.86 | Very high risk |
| N1 | 196 | 0.27 | 441 | 0.70 | 2.45 | 0.35 | 1.225 | 4.61 | Very high risk |
| N2 | 140 | 0.17 | 302 | 0.50 | 0.76 | 0.25 | 0.38 | 3.42 | High risk |
| N3 | 251 | 0.25 | 870 | 0.60 | 0.63 | 0.3 | 0.315 | 4.32 | Very high risk |
| N4 | 235 | 0.20 | 479 | 0.56 | 0.71 | 0.28 | 0.355 | 3.94 | High risk |

* These are ratios between the measured concentrations, and the concentrations based on accepted concentrations in aquatic ecosystem health.
