# Supplementary material for: Agricultural intensification in Lake Naivasha Catchment in Kenya and associated nutrients and pesticides pollution
Source: Sci Rep. 2024 Aug 9;14:18539. doi: 10.1038/s41598-024-67460-5 (PMC11315982; doi:10.1038/s41598-024-67460-5)
Supplement: Supplementary file 3 — Supplementary Table 3. [file 41598_2024_67460_MOESM3_ESM.docx]

**Supplementary Table 3: Water quality standard for the protection of aquatic life.**

| **Contaminant of reference** | **USEPA guideline^1^ value**  **(µg/L)** | **Kenya effluent standards^2^ into aquatic environment (µg/L)** |
| --- | --- | --- |
| Sum DDT | 0.0015 | *-* |
| Sum HCH | 0.95 | - |
| Sum Cyclodienes | 0.004 | - |
| Total nitrogen | 1000 | 2000 |
| Total phosphorus | 67 | 2000 |

*Source: Based on values documented by US EPA* (2014) *and WASREB* (2008)*.*

^1^ – Guideline values are considered as indicative and not legally bindings. As used in this study, they indicate the limites, beyond which concentratiions may affect the quality of aquatic flora and fauna.

^2^ – Standards are considerd a legaly binding and there is a risk of prosecution people do not adhere to the standards. In these study, these values are drawn from legal authorities responsible for protection of aquatic life.
