# Supplementary material for: Agricultural intensification in Lake Naivasha Catchment in Kenya and associated nutrients and pesticides pollution
Source: Sci Rep. 2024 Aug 9;14:18539. doi: 10.1038/s41598-024-67460-5 (PMC11315982; doi:10.1038/s41598-024-67460-5)
Supplement: Supplementary file 4 — Supplementary Table 4. [file 41598_2024_67460_MOESM4_ESM.docx]

**Supplementary Table 4: Decadal land use change in the Lake Naivasha catchment from 1989 to 2019 (in km^2^)**

| Year | Water | Evergreen Broadleaf Forest | Cropland | Grasslands | Bare Soils | Built-Up |
| --- | --- | --- | --- | --- | --- | --- |
| 1989 | 117 | 404 | 293 | 531 | 1,299 | 47 |
| 1999 | 133 | 454 | 637 | 660 | 1,706 | 84 |
| 2009 | 102 | 266 | 277 | 803 | 1,271 | 234 |
| 2019 | 144 | 215 | 915 | 1,065 | 1,008 | 588 |
