# Supplementary material for: Agricultural intensification in Lake Naivasha Catchment in Kenya and associated nutrients and pesticides pollution
Source: Sci Rep. 2024 Aug 9;14:18539. doi: 10.1038/s41598-024-67460-5 (PMC11315982; doi:10.1038/s41598-024-67460-5)
Supplement: Supplementary file 5 — Supplementary Table 5. [file 41598_2024_67460_MOESM5_ESM.docx]

**Supplementary Table 5: Probability of intensified agricultural expansion among the sub-catchment in L. Naivasha.**

| **Sub-catchment** | **1999** | **2009** | **2019** |
| --- | --- | --- | --- |
| K1 | Semi-intensification | Extensive | Extensive |
| G1 | Semi-intensification | Full intensification | Full intensification |
| G2 | Extensive | Extensive | Extensive |
| G3 | Semi-intensification | Semi-intensification | Extensive |
| M1 | Semi-extensive | Full intensification | Full intensification |
| M2 | Semi-extensive | Full intensification | Extensive |
| M3 | Semi-intensification | Semi-intensification | Semi-extensive |
| M4 | Semi-intensification | Full intensification | Semi-extensive |
| M5 | Semi-intensification | Full intensification | Semi-extensive |
|  |  |  |  |
| Lake area | Semi-extensive | Semi-intensification | Semi-intensification |
