# Supplementary material for: Agricultural intensification in Lake Naivasha Catchment in Kenya and associated nutrients and pesticides pollution
Source: Sci Rep. 2024 Aug 9;14:18539. doi: 10.1038/s41598-024-67460-5 (PMC11315982; doi:10.1038/s41598-024-67460-5)
Supplement: Supplementary file 6 — Supplementary Table 6. [file 41598_2024_67460_MOESM6_ESM.docx]

**Supplementay Table 6: Coefficient results of the multinomial linear regression with Extensive intensification level as reference class**

| Level of expansion* | (Intercept) | TN | TP | ∑DDT | ∑HCH | ∑cyclodienes |
| --- | --- | --- | --- | --- | --- | --- |
| Full intensification | 3.351 | 0.079 | -0.141 | -0.323 | 0.060 | 0.016 |
| Semi-intensification | -2.221 | -0.040 | -0.005 | 0.079 | 0.158 | 0.017 |
| Semi-extensive | 4.198 | -0.081 | 0.101 | 0.225 | -0.080 | 0.016 |
| Relatively natural | 1.142 | 0.015 | -0.076 | -0.214 | 0.175 | 0.032 |
| *Agricultural expansion results to increased heterogeneity, reduced forest cover, increased cropland, and reduced grassland | | | | | | |
